# Assessing the expression of immunosuppressive cytokines in the newly diagnosed systemic lupus Erythematosus patients: a focus on B cells

**DOI:** 10.1186/s12865-020-00388-3

**Published:** 2020-11-16

**Authors:** Mitra Abbasifard, Zahra Kamiab, Mohammad Hasani, Amir Rahnama, Pooya Saeed-Askari, Hossein Khorramdelazad

**Affiliations:** 1 Department of Internal Medicine, Ali-Ibn Abi-Talib Hospital, School of Medicine; Molecular Medicine Research Center, Research Institute of Basic Medical Sciences, Rafsanjan University of Medical Sciences, Rafsanjan, Iran; 2 Clinical Research Development Unit, Ali-Ibn Abi-Talib Hospital, Rafsanjan University of Medical Sciences, Rafsanjan, Iran; 3 Department of Family Medicine, School of Medicine, Rafsanjan University of Medical Sciences, Rafsanjan, Iran; 4 Department of Pathology, School of Medicine, Rafsanjan University of Medical Sciences, Rafsanjan, Iran; 5 Department of Immunology, School of Medicine; Molecular Medicine Research Center, Research Institute of Basic Medical Sciences, Rafsanjan University of Medical Sciences, Rafsanjan, Iran

**Keywords:** Regulatory B-cells (Bregs), Systemic lupus Erythematosus (SLE), Anti-inflammatory cytokine

## Abstract

**Background:**

The immunosuppressive effects of regulatory B-cells (Bregs) and their immunosuppressive cytokines on immune responses in autoimmune disorders, mainly systemic lupus erythematosus (SLE), have been recently established. Therefore, the purpose of this article has been the exploration of the expressions of cytokines produced by B cells in newly diagnosed SLE patients.

**Results:**

The findings demonstrated that the gene expression of IL-10, TGF-β, IL-35, PD-L1, and FasL was significantly up-regulated in SLE patients compared to healthy subjects (*P* < 0.05). Additionally, the results revealed that serum levels of IL-10, TGF-β, IL-35, PD-L1 were remarkably increased in patients with SLE compared to healthy subjects (*P* < 0.0001). However, serum levels of IL-10 and TGF-β decreased significantly with increasing SLEDAI score in studied patients (*P* < 0.05).

**Conclusion:**

It was concluded that the release of anti-inflammatory cytokines, particularly IL-10 and TGF-β, might inhibit immune responses and autoreactive immune cells in a compensatory manner in SLE patients with mild to moderate disease activity.

## Background

The systemic lupus erythematosus (SLE) is a multifactorial chronic autoimmune disease that is more common in women [1]. Evidence showed that genetic and environmental factors, including cigarette smoking, drugs, ultraviolet (UV) light, chemical substances, gut microbiota, and viral infections, could be involved in SLE onset [1, 2]. Regarding the existence of an imbalance between apoptosis and removal of apoptotic substances in SLE patients, nuclear antigens such as histones, centromere proteins, single- and double-stranded deoxyribonucleic acid (ss- and ds-DNA), nucleosome, Smith antigen (Sm Ag), Ro and La proteins, as well as ribonucleoproteins (RNPs) become exposed to the immune system cells and components [3]. These autoantigens are able to stimulate B-cells to produce auto-antibodies such as anti-nuclear antibody (ANA) and anti-double-stranded DNA (ds-DNA) antibody as well as other inflammatory mediators [4].

Previous studies revealed that regulatory immune cells, such as regulatory T cells (Tregs) and B regs with immunosuppressive properties, are involved in the homeostasis processes [5]. Recent experimental and human studies have further indicated that Bregs can suppress inflammatory responses via production and secretion of anti-inflammatory cytokines like IL-10, IL-35, and TGF-β as well as expression of inhibitory molecules [6, 7]. Besides, Bregs come up from common progenitor of transitional 2-marginal zone precursor (T2-MZP) B cells capable of being autoreactive following interaction with pathogens and even activating release anti-inflammatory mediators [8]. Some investigations in this respect have correspondingly suggested that Bregs are impaired in inflammatory autoimmune disorders such as SLE, rheumatoid arthritis (RA), and Graves’ disease [9]. However, signals necessary for differentiation of Bregs have remained poorly understood, and previous studies have revealed that, under normal conditions, plasmacytoid dendritic cells (pDCs) would stimulate differentiation of CD19 + CD24^hi^CD38^hi^ immature B-cells into the CD24^+^CD38^hi^ mature Bregs, which could produce IL-10 through release of interferon-alpha (IFN-α) and the cluster of differentiation 40 (CD40) engagement. Conversely, the mentioned Bregs can have an inhibitory effect on the generation of IFN-α through pDCs by releasing IL-10 [10]. In SLE, the cross-talk between pDCs and Bregs has defected, and pDCs fail to trigger differentiating CD19^+^CD24^hi^CD38^hi^ B-cells into IL-10-producing CD24^+^CD38^hi^ Bregs [10]. In addition, Bregs can suppress CD4^+^ T-cell proliferation, induce Foxp3^+^ regulatory T and Tr1 cells, and suppress T helper (Th) 1 (Th1). Bregs also can stimulate Th17 and CD8^+^ effector T-cells differentiation through cell-cell interactions and release of the anti-inflammatory cytokine, including TGF-β, IL-35, and IL-10. As well, TGF-β and IL-10, which are secreted by Bregs, can have an inhibitory effect on antigen-presenting function, cytokine secretion by DCs, neutrophils, natural killer (NK) cells, M1 macrophages, and conversely, induction of M2 macrophage differentiation.

Moreover, FasL (or CD178) or PD-L1 (or CD274) that are expressed on the Bregs surface are involved in the apoptosis of effector T cells following ligation with PD-1 (CD279) and Fas (CD95) on the surface of mentioned T-cells [11]. Since changes in immune cells phenotype, as well as their number and function, are closely related to the disease activity, measuring the produced mediators and inhibitory molecules by regulatory immune cells before treatment based on diseases activity status could be critical in further understanding the role of the modulatory mechanisms of the immune system in the pathogenesis of SLE. Therefore, this study aimed to measure inhibitory molecules’ expression on B cells’ surface and anti-inflammatory cytokines produced by B cells in newly diagnosed SLE patients compared to healthy subjects.

## Results

Twenty-three patients suffering from SLE and thirty normal-age and gender-matched subjects were enrolled in this study. The demographic features of SLE patients and healthy subjects are shown in Table 1. All the patients were classified as individuals having an active disease (SLEDAI ≥4). The mean ± SD of SLEDAI in patients was 9.73 ± 6.01and we divided patients into three groups (SLEDAI I; 4 to 7, SLEDAI II; 7 to10, SLEDAI III more than 10) based on SLEDAI scores (min:5, max: 34, and median: 8). At the time of the blood collection, newly diagnosed SLE patients were not under treatment. Clinical and laboratory data of studied SLE patients are shown in Table 2.

**Table 1:** Some demographic features of patients and healthy subjects enrolled in the study

Demographic features	Control *n* = 30	Patients *n* = 23	*P*-Value
Age at study (years), mean ± SD	34.95 ± 10.02	39.60 ± 13.45	0.21
Sex (m/f)	0/30	0/23	–
BMI (kg/m2), mean ± SD	23.87 ± 2.97	26.97 ± 4.95	0.019*
Disease activity (SLEDAI), mean ± SD	–	9.73 ± 6.01	–
Smoking (%)	0 (0)	0 (0)	–

Data are presented as mean ± SD, * significant (*P* < 0.05)

**Table 2:** Clinical and laboratory data of studied SLE patient

SLE patient (n = 23)	WBC/mm^**3**^	PLT/(μL)	Anemia	Proteinuria	CRP (mg/mL)	ESR (mm/hour)	RF (IU/mL)	ANA	Anti-dsDNA	Anti-SSA-Ro	Lupus anticoagulant antibody	Anti-cardiolipin antibody	C3/C4 deficiency	Seizure	Arthritis	Serositis	Alopecia	Oral ulcer	Skin lesion	Photosensitivity
1	5100	327,000	–	–	10.5	26	64	+	–	+	–	–	–	–	+	–	+	–	+	+
2	4400	430,000	–	–	11.8	27	11.4	+	–	–	–	–	–	–	+	–	+	–	+	+
3	2900	200,000	–	+	5.1	7	8.6	+	+	–	–	–	–	–	+	+	+	+	–	+
4	6300	205,000	–	–	4.3	14	19.4	+	–	–	+	–	+	–	+	–	–	–	+	+
5	6900	431,000	–	–	6.8	13	12.6	+	+	–	–	–	–	–	+	–	–	–	+	+
6	4200	431,000	–	–	10.5	10	29.5	+	–	–	–	–	–	–	+	+	–	–	+	+
7	6300	341,000	–	–	13.2	5	17.4	+	+	–	–	–	–	–	+	–	+	–	+	+
8	4400	384,000	–	–	5.5	9	9.09	+	–	+	–	–	–	–	+	–	+	–	+	+
9	3100	421,000	–	–	6.5	4	6.71	+	+	–	+	–	–	–	+	+	+	–	+	+
10	5700	293,000	–	–	7.1	4	5.3	–	+	–	–	–	–	–	+	–	+	–	+	+
11	9400	230,000	–	–	3.2	4	4.1	+	+	–	–	–	–	–	+	–	–	–	+	+
12	8400	432,000	–	–	6.4	26	16.2	+	–	–	+	–	–	–	+	–	–	+	+	+
13	9600	305,000	–	–	0.4	5	2.7	+	+	–	–	–	–	–	+	–	–	–	+	+
14	5700	393,000	–	–	15.2	58	14.3	+	+	–	–	–	+	–	+	–	–	–	+	+
15	7500	287,000	–	–	18.3	47	128.8	+	–	–	–	–	–	–	+	–	–	–	+	+
16	3300	375,000	+	–	9.7	6	5.1	+	+	–	–	–	–	–	+	–	+	–	_	+
17	4800	447,000	–	–	4.5	8	9.2	+	+	–	–	–	–	–	+	–	+	–	+	+
18	7100	307,000	–	–	13.1	17	8.8	+	–	–	–	–	–	–	+	–	+	–	–	–
19	7000	387,000	–	–	14.1	32	129.6	–	–	+	–	+	+	–	+	–	–	–	+	+
20	8900	270,000	–	–	6.1	26	14.5	+	+	–	–	–	–	+	+	–	+	+	+	+
21	5200	422,000	–	–	8.4	18	22.1	+	–	–	–	–	–	–	+	–	–	–	+	+
22	6900	394,000	–	–	10.8	14	8.41	+	–	–	–	–	–	–	+	+	+	–	+	+
23	3500	110,000	–	–	3.7	24	6.7	+	+	–	–	–	–	–	+	–	+	–	+	+
All (mean ± SD) or percentage	**6335 ± 1811**	**340,087 ± 91,498**	**4.3%**	**4.3%**	**8.4±** **4.4**	**17.5 ± 14.2**	**24.1 ± 35.4**	**91.3%**	**52.17%**	**13%**	**13%**	**4.3%**	**13%**	**4.3%**	**100%**	**17.3%**	**56.5%**	**13%**	**86.9%**	**95.6%**

### mRNA and protein expression of cytokines and inhibitory molecules

**Fig. 1:**
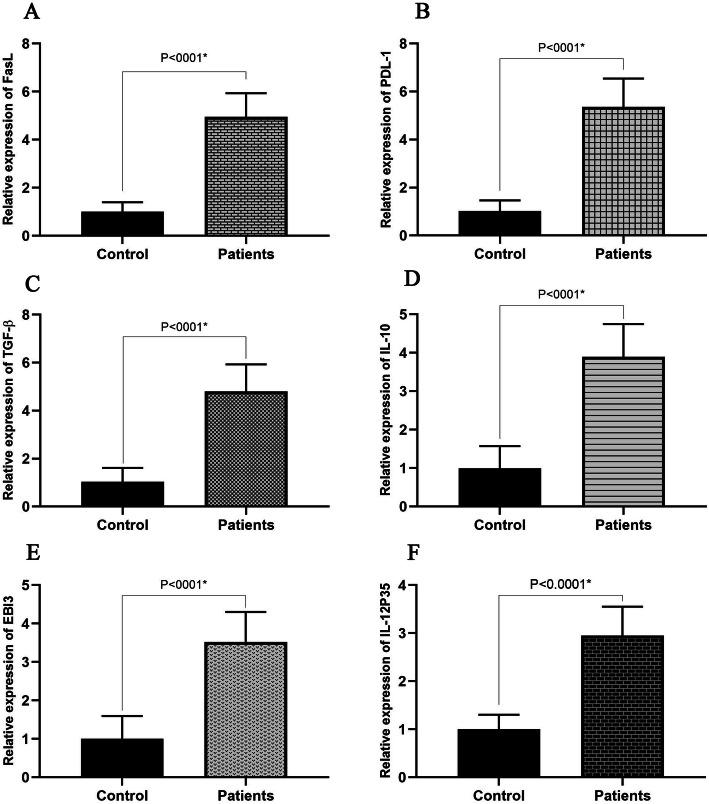
Alteration in mRNA levels of FasL (**a**), PDL-1 [2], TGF-β (**c**), IL-10 (**d**), EBI3 (**e**), and IL-12p35 (**f**) in control and SLE patient groups. Data are presented as mean ± SD, * significant (*P* < 0.05)

**Fig. 2:**
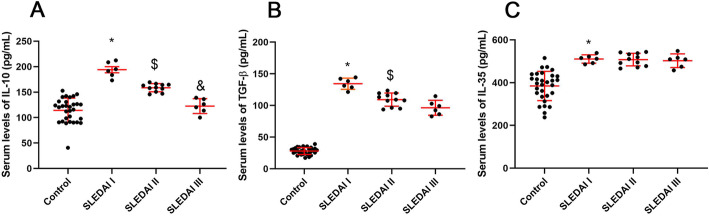
The differences between serum levels of IL-10 (**a**), TGF- β (**b**), and IL-35 (**c**) in healthy subjects (control) and three groups of SLE patients based on SLEDAI I; 4 to 7, SLEDAI II; 7 to10, SLEDAI III more than 10 scores. *: significant difference between control and three SLEDAI groups, $: significant difference between SLEDAI I and SLEDAI II groups, &: significant difference between SLEDAI I, SLEDAI II, and SLEDAI III groups. Data are presented as mean ± SD, * significant (*P* < 0.05)

The data obtained from RT-PCR demonstrated the considerable rising trend in expressions of IL-10, IL-35 (EBI3 or IL-12P35), TGF-β, PDL-1, and FasL genes (*P* < 0.0001) in the B-cell population of the SLE patients compared with ones isolated from the healthy subjects (Fig. 1). Additionally, serum levels of IL-10 (control: 115 ± 22.1; patient: 158 ± 31.7; *P* < 0.001), IL-35 (control: 377 ± 63.84; patient: 499 ± 42.95; P < 0.001), and TGF-β (control: 28 ± 6.87; patient: 112 ± 19.47; *P* < 0.005) were significantly increased in SLE patients compared with the healthy subjects. The multiple comparison analysis in three groups of patients based on the SLEDAI score showed that by increasing disease activity, serum levels of IL-10 and TGF-β decreased significantly (*P* < 0.05), while in the case of IL-35, a significant difference was not observed. Serum levels fluctuations in SLE patient different SLEDAI scores are shown in Fig. 2.

### Cytokines, autoantibodies, inhibitory molecules and disease activity

**Fig. 3:**
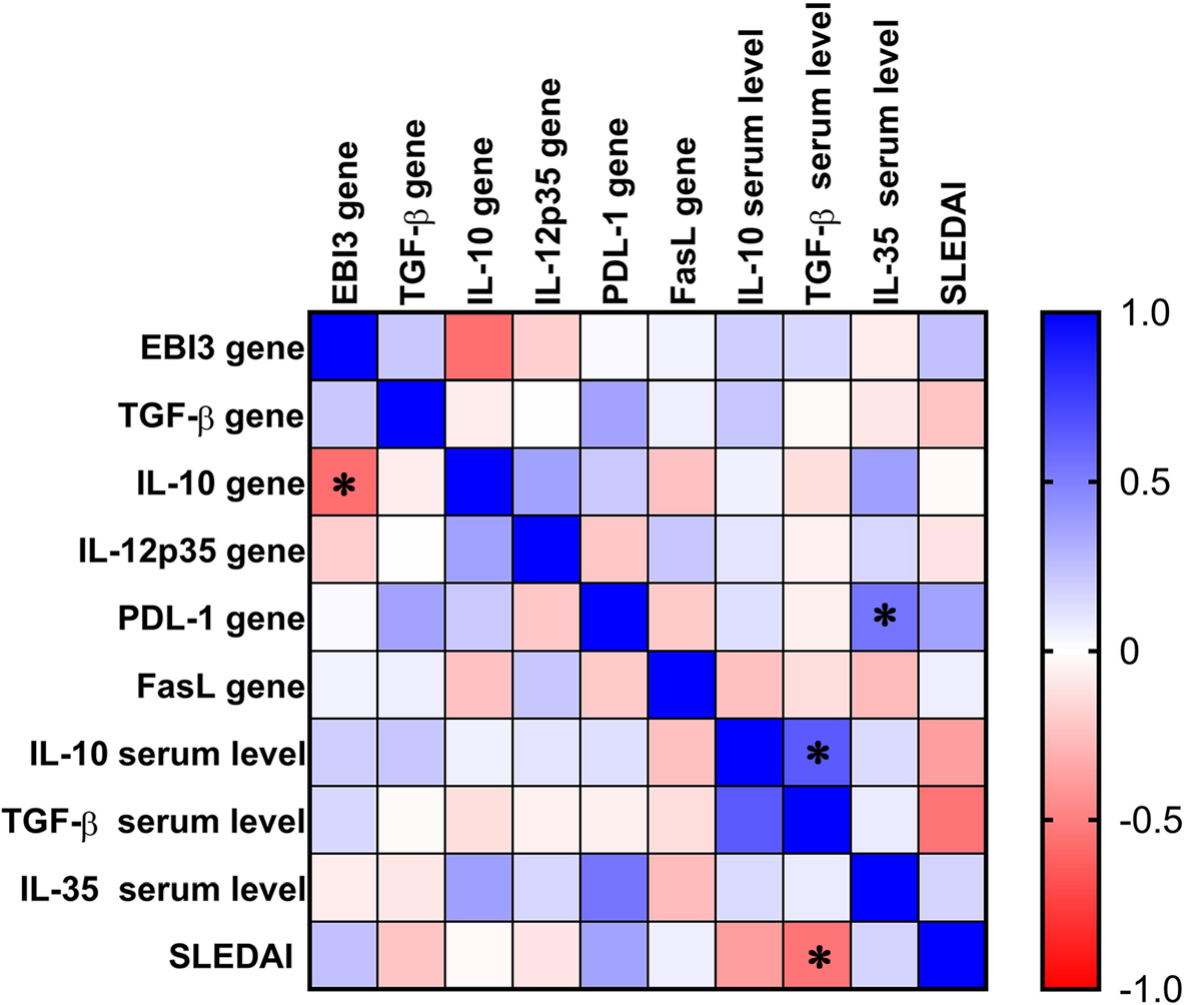
The correlations between SLEDAI as the disease activity index in SLE patients and studied inhibitory molecules as well as immunosuppressive cytokines. The confidence interval of r is shown in the right rectangle (between − 1 and 1). * significant (*P* < 0.05)

The correlation analysis between disease activity scores and titres of autoantibodies showed that there was a positive association between ANA, anti-dsDNA, and anti-SSA/Ro with disease activity score but the correlations were not statistically significant (r = 0.2, *p* = 0.359; r = 0.108, *p* = 0.623; and r = 0.353, *p* = 0.1; respectively). Correlation matrix analysis showed that there was an association between serum level of TGF-β and SLEDAI (r = − 0.54, *p* = 0.007); PDL-1 gene and IL-35 serum level (r = 0.53, *p* = 0.008); IL-10 and TGF-β serum levels (r = 0.64, *p* = 0.001); IL-10 and EBI3 genes (r = − 0.56, *p* = 0.05) (Fig. 3).

**Fig. 4:**
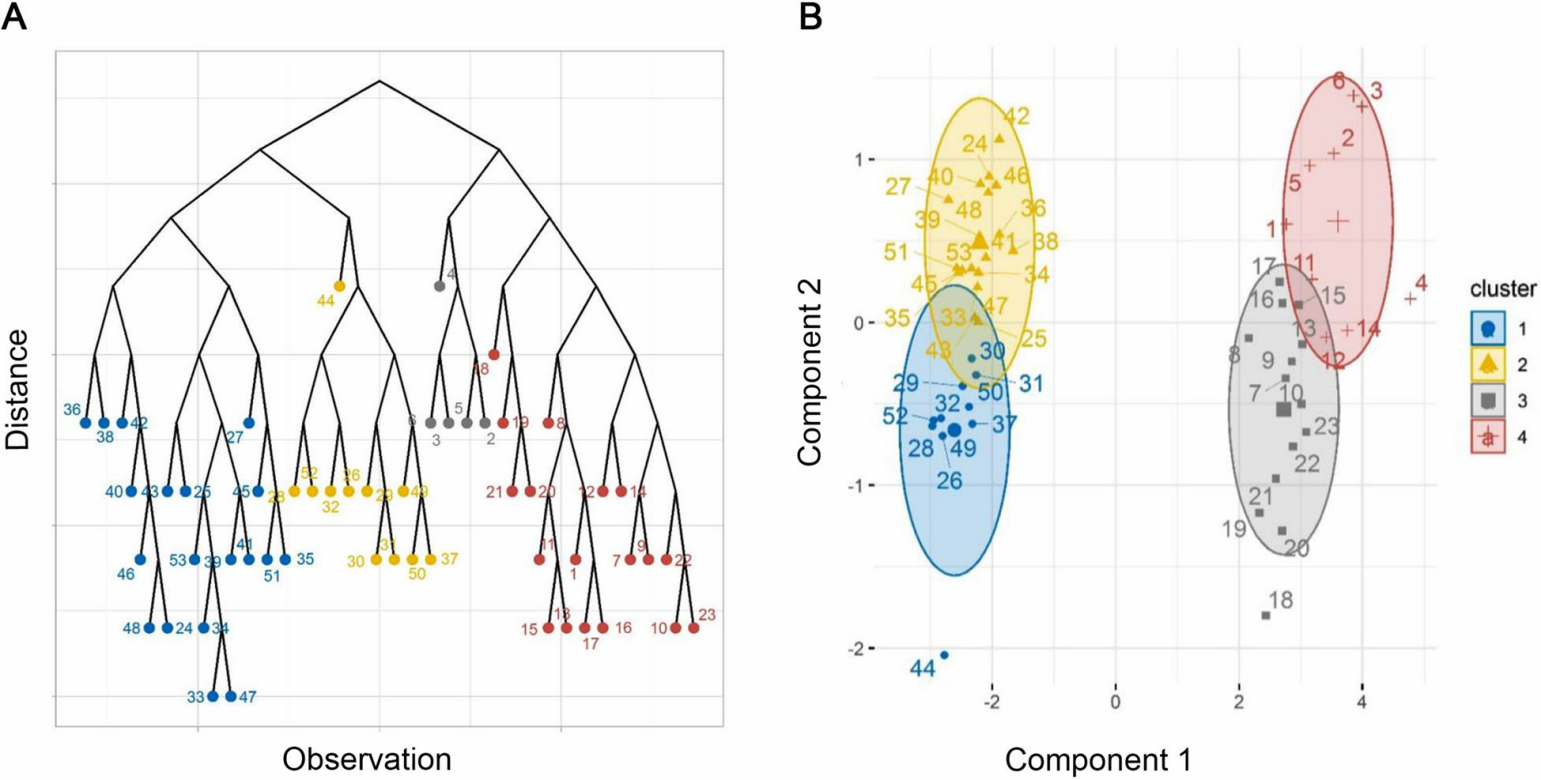
Principal component analysis and clustering of cytokine profiles in newly diagnosed SLE patients (*n* = 23) and healthy subjects (*n* = 30). Information of nine variables related to studied cytokines (EBI3, TGF-β, IL-10, IL-12p35, PDL-1, FasL genes and IL-10, TGF-β, and IL-35 serum levels) were extracted and converted into two components 1, and 2 using PCA based on the loading values. The cumulative percent of the variance of the two components was 88.47. Data showed that healthy subjects were placed in clusters 1 and 2 (blue and yellow) and to the left of the dendrogram, while SLE patients were placed in clusters 3 and 4 (red and gray) and to the right of the dendrogram (**a**). The results also showed that by considering components 1 and 2 data extracted from the mRNA and protein levels of IL-10, TGF-β, and IL-35, SLE patients could be differentiated from healthy subjects (**b**)

Moreover, information of nine variables related to studied cytokines (EBI3 gene, TGF-β, IL-10, IL-12p35, PDL-1, FasL genes and IL-10, TGF-β, and IL-35 serum levels) were extracted and converted into two components 1, and 2 using PCA. The cumulative percent of the variance of the two components was 88.47%. HCA was also used to cluster patients based on cytokine obtained data. Data showed that healthy subjects (*n* = 30) were placed in clusters 1 and 2 (blue and yellow) and to the left of the dendrogram while SLE patients (*n* = 23) were placed in clusters 3 and 4 (red and gray) and to the right of the dendrogram (Fig. 4 a). Altogether, the results showed that considering components 1 and 2 data, SLE patients could be differentiated from healthy subjects (Fig. 4 b).

Additionally, the results of regression (PCR) showed that there is only a significant inverse relationship between component 2 and SLEDAI scores because the estimated confidence interval for component 2 did not include zero; therefore, on average, with the increase of one unit of component 2 in which IL-10 plays an important role, the SLEDAI score has decreased 1.39 units (Table 3). These results suggest that there is an inverse association between SLEDAI scores and immunomodulatory cytokines.

**Table 3:** bootstrap linear regression with 95% bias-corrected confidence interval (CI) for evaluation of the association between SLEDAI scores and cytokines adjusted to auto-antibodies in SLE patients

Variables	Estimate	95%CI
		(Lower, Upper)
**(Intercept)**	9.936	(6.444, 36.227)
**Component 1**	−0.438	(−1.468, 4.987)
**Component 2**	−1.399	(−3.089, −0.458) *
**Anti dsDNA (Positive)**	0.000	(−1.358, 1.920)
**ANA (Positive)**	−0.186	(−13.723, 1.490)
**Anti-SSA/Ro (Positive)**	−0.854	(−2.174, 11.219)

Components 1 and 2 are related to cytokines, * significant association

## Discussion

In this study, the expression of some inhibitory molecules and immunosuppressive cytokine in isolated B cells and sera from newly diagnosed SLE patients whose treatment had not been started was investigated. The study findings revealed that mRNA levels of IL-35 (EBI3 or IL-12p35), IL-10, and TGF-β in isolated B-cells from SLE patients were elevated compared to healthy subjects. Moreover, the level of IL-10, TGF-β, and IL-35 serum increased in the patients’ peripheral blood affected by the SLE compared with healthy subjects. The findings in three groups of patients based on SLEDAI score also demonstrated that by increasing disease activity, serum levels of IL-10 and TGF-β decreased significantly (*P* < 0.05), although, in the case of IL-35, there was no remarkable difference between groups. Additionally, the mRNA levels of PDL-1 and FasL were significantly up-regulated in B-cells of the SLE patients compared to healthy subjects.

Evidence revealed that SLE is a multiple-organ autoimmune disease described by autoantibodies’ increased production against autoantigens and enhanced immune complex deposition. Both T- and B-cells with different phenotypes are also involved in SLE’s pathogenesis [12]. Previous studies on a subset of B cells with modulatory properties in patients with RA, primary Sjogren’s syndrome (SjS), and SLE have demonstrated a hyperfunction in these B cells anti-inflammatory cytokines like IL-10 production and eventually homeostasis [9, 13, 14]. Several investigations have shown that depletion of pan B cell could help SLE patients, while phase III trials of the pan-B cell depletion were unsuccessful [15]. This problem can be due to the depletion of both the effector and regulatory B cell subsets [6]. These findings could accordingly confirm the immune-modulatory properties of Bregs and their involvement in the homeostasis mechanisms [16]. The first clinical investigation of peripheral blood B cells in SLE patients revealed that the proportion of CD5^+^ B cells producing IL-10 was significantly higher than healthy subjects [17]. Although there are several contradictions in this field, some studies have shown that the number of Bregs in autoimmune diseases such as RA and SLE decreased [13]; on the other hand, some experimental and human studies have demonstrated that the number of Bregs was increased in SLE which can ultimately depend on various factors such as treatment protocol and disease activity. In this study, the results show that apart from the number, a group of B cells might be hyperactivated to cause homeostasis in patients with lower SLEDAI scores [17–21]. In line with our findings, it was shown that in humans, IL-10^+^ B cell frequency in SLE patients was higher than the healthy control group [17, 20]. These findings also confirm the possibility of hyperactive Bregs involvement in balancing inflammatory and anti-inflammatory responses. Although mechanisms that mediate development and induction of the Bregs have thus far remained unclear, a study suggested that the increased IL-10^+^ Bregs may be secondary to expanded T follicular helper (Tfh) cells provide help to effector B cells and promote autoimmunity. Tfh cell and IL-10^+^ Bregs are increased in SLE and Tfh cell-derived IL-21 induced IL-10 [22]. In most human studies that have been done so far, SLE patients were under different treatment protocols, affecting the outcomes [13]. For instance, methotrexate can increase the number and possibly, the activity of B cells [23]. Another study on murine collagen-induced arthritis showed that treating animals with methotrexate, alone or together with cyclophosphamide, can reduce Bregs and DCs in lymph nodes and spleen [24].

In contrast, another investigation reported no associations between azathioprine, methotrexate, mycophenolate mofetil, and hydroxychloroquine with the expression of IL-10. However, the mentioned study showed that the serum level of IL-10 was twice more in SLE patients with Asian ethnicity than non-Asians [25]. The results of this study show that patients’ ethnicity can also affect the level of anti-inflammatory cytokines. Recently it has been shown that systemic treatment with methotrexate may cause dysregulation of anti-inflammatory cytokines [26]. According to the mentioned studies, it seems that treatment with immunomodulatory drugs can change the cytokine profile and negatively affect the results. In our study, the results showed that in newly diagnosed SLE patients with mild or moderate disease activity who were not under any treatment, anti-inflammatory cytokines as well as inhibitory molecules (as suppressive indicators of B cells) expression increased by isolated B cells in compared to healthy subjects and by increasing the SLEDAI score, serum levels of IL-10 and TGF-β decreased remarkably. However, the balance between regulatory and effector functions is a finely regulated immunological process that is not fully understood.

In this study, it was observed that IL-35 levels significantly elevated in SLE patients compared to healthy subjects. In this regard, an investigation reported that IL-35 could induce B cells and stimulate their differentiation into a regulatory subset producing IL-35 and IL-10. These findings similarly proposed that IL-35 is a potential inducer of the autologous and IL-35^+^ B cells in treating inflammatory and autoimmune diseases [27]. It had been further confirmed that circulatory IL-10^+^ Bregs had significantly increased in SLE patients, accompanied by fluctuation such that the number and activity of the Bregs had elevated during SLE flares and reduced subsequent remission of the disease [22]. Our findings also showed that as the SLEDAI score increased, the levels of IL-10 and TGF-β significantly decreased in studied SLE patients, although no decrease was observed in the serum level of IL-35. These findings could confirm the role of disease activity in the fluctuation of the B cells’ number and activity in SLE patients.

It should be noted that myeloid-derived suppressor cells (MDSCs) as an immune suppressor cell can also induce the development of Bregs through inducible nitric oxide synthase (iNOS) and relieve self-immune responses in a lupus animal model [28]. Moreover, Bregs are not the only source of immunosuppressive cytokines because other cells might produce and release IL-10, TGF-β, and IL-35 in response to inflammatory and pathological conditions to modulate the immune response. Evidence showed that the regulatory T cells (Tregs) and Bregs involvement in different phases of autoimmune diseases could be different. For instance, a study had shown that IL-10^+^ Bregs could mainly regulate the initiation phase of the disease, while Tregs could cooperatively inhibit late-phase via covering IL-10^+^ Bregs in an experimental autoimmune encephalomyelitis (EAE) model of multiple sclerosis (MS) disease [29].

Berthelot et al. had reported that induction of apoptosis by Bregs through PD-1, FasL, or TNF-related apoptosis-inducing ligand (TRAIL) differed with the nature of the target T cell. T-cell subsets’ sensitivity probably shifted from Th1 and Th2 to Th17, resulting in a reduction in Tregs [30]. The outputs of this study also demonstrated that the expressions of the PDL-1 and FasL gene had significantly elevated in isolated B-cells of the patients with SLE compared to healthy subjects. It could be another mechanism of B cell for modulating the immune response by eliminating effector immune cells.

On the other hand, some studies found that regulatory immune cells’ function to inhibit effector T cells in inflammatory and autoimmune illnesses like lupus was defective. However, in most of these investigations, serum levels of IL-35, IL-10, and TGF-β cytokines along with PDL-1 and FasL had not been measured in B cells. The wide range of disease activity in SLE patients and treatment protocols might lead to these discrepancies in the studies’ findings.

Our findings showed a negative association between serum level of TGF-β and SLEDAI; IL-10 and EBI3 genes may be due to reduced Bregs function in patients with higher disease activity scores. However, a positive and significant correlation was observed between the PDL-1 gene and serum level of IL-35, as well as IL-10 and TGF-β serum levels [7]. Furthermore, regarding the studied cytokines’ extracted data at mRNA and protein levels (components 1 and 2), clustering showed that it is possible to differentiate patients from healthy subjects using the information in components 1 and 2. Additionally, statistical analysis demonstrated that with increasing the SLEDAI score, component 2 was significantly decreased. Therefore, with increasing disease activity, the level of immunomodulatory cytokines produced by B cells in patients with SLE might significantly be decreased.

One of the strengths of this study was that all the patients enrolled in the study were newly diagnosed cases, and blood collection was done before treatment onset because, as discussed before, routine treatment for lupus patients could affect the number and activity of B cells and outcome of the studies [17]. The inaccessibility of the materials for fluorescence-activated cell sorting (FACS) technique to specific isolation and evaluation cytokine levels in Bregs was also declared one of the major limitations of this study.

## Conclusion

Taken together, the results of this study indicated that a group of SLE patients B cells might modulate immune responses in mild to moderate disease activity by producing anti-inflammatory cytokines and expressing inhibitory molecules. The findings can also clarify SLE disease activity’s effect on the fluctuation and expression of IL-10, TGF-β serum levels. However, the source of the studied cytokines might be other regulatory immune cells. Future studies are needed to fully elucidate the source of released anti-inflammatory cytokines and evaluate the producer regulatory cells’ number and activity to a well understanding of the immunomodulatory mechanisms in SLE.

## Methods

### Subjects

Twenty-three newly diagnosed SLE patients (mean age of 39.6 ± 13.4 years) diagnosed by a rheumatology specialist and 30 normal-age and gender-matched healthy subjects with a mean age of 34.95 ± 10.01 years have participated in this study. All SLE patients had at least four of the American College of Rheumatology (ACR) revised SLE criteria. Moreover, activities of the SLE disease have been evaluated through Systemic Lupus Erythematosus Disease Activity Index (SLEDAI), and all the patients showed active SLE (SLEDAI score > 4). All of the enrolled SLE patients had received no treatment up to the blood collection for the experiments. Additionally, No SLE patient has been receiving immunomodulatory medications include antimalarials, at the time of examination. Informed consent was received from each SLE patient and healthy subjects based on Declaration of Helsinki (DoH). Moreover, this investigation was approved by the Ethics Committee of Rafsanjan University of Medical Sciences, Rafsanjan, Iran (IR.RUMS.REC.1389.206).

### Cytokine assay

Five mL of peripheral blood was obtained from the studied groups, and separation of serums was performed by low-speed centrifugation. Then, serum samples were kept at − 20 °C for further experiments. Next, ELISA kits were used in order to measure circulatory levels of anti-nuclear antibody (ANA) (TECAN, IBL, Hamburg, Germany), IL-10 (Abnova, KA0125, Taiwan), IL-35 (Cosabio, CSB E13126h, Wuhan, China), and TGF-β (Abnova, KA3108, Taiwan) based on the assay procedures documented in the users’ instructions manual provided by the manufacturers. The coefficients of variation of intra- and inter-assays were 5 and 15%, respectively.

### Isolation and storage of peripheral blood mononuclear cells (PBMCs)

First, to isolate peripheral blood mononuclear cells (PBMCs), peripheral blood samples were gently diluted in PBS. Next, PBMCs were isolated using Ficoll (Lymphodex, Inno-Train, Germany). For further experiments, fresh PBMCs were utilized.

### B cells isolation

A nylon wool fiber (NWF) procedure using nylon woolpack (Polysciences Inc., USA) was employed to separate B cells. Briefly, the column washed with the media contained Dulbecco’s phosphate-buffered saline (DPBS) consisting of 5% heat-inactivated fetal calf serum (FCS), RPMI-1640 with 10% FCS, Hank’s balanced salt solution (HBSS) consisting of 10% FCS, McCoy’s 5A medium with 10% FCS at 37 °C, Earle’s saline (ES) containing 10% FCS, and prepared column incubated for one hour at 37 °C. Next, 1 × 10^8^ viable PBMCs were added to each column in a volume of 2 ml of the media. The stop-cock was also opened and allowed the media for draining until the cell volume entered into the packaged wool. The column top was then further washed with an additional 2 ml of the media and allowed the washed column to enter the packaged wool. The other 2–5 ml of the media was added to the column for ensuring that the wool top had been covered with the medium, and it was then incubated for one hour at 37 °C. Besides, nonadherent T-cells have been collected by two washes. Afterward, adherent B-cells were collected by adding the media for filling the column as well as knocking it to dislodge the cells. Therefore, this column was plunged twice, and the collected cells spun down at 1200 rpm for ten minutes, and the supernatant has been removed. Finally, the cell pellet has been re-suspended in approximately 10 ml of the media.

### Flowcytometry

**Fig. 5:**
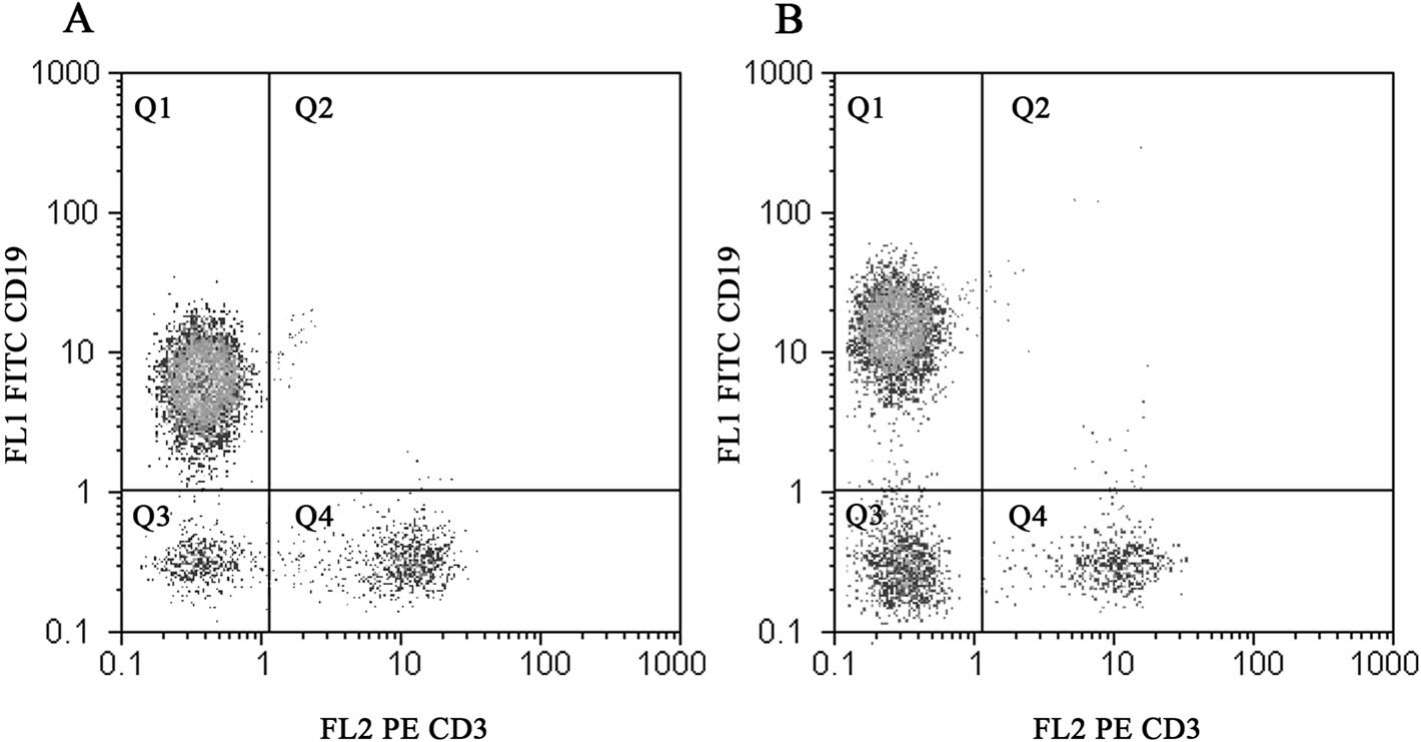
The expression of CD19+ B cells in healthy subjects (**a**) and SLE patients (**b**). Isolated B cells flow cytometry analysis: CD19^+^/CD3^−^(Q1, A; 82.97 ± 9.9%, B; 80.14 ± 5.7%); CD19^+^/CD3^+^ (Q2, A; 0.9 ± 0.12%, B;1.23 ± 0.27%); CD19^−^/CD3^−^ (Q3, A; 9 ± 2.5%, B;10.7 ± 3.26%); CD19^−^/CD3^+^ (Q4, A;7.13 ± 2.1%, B; 7.93 ± 1.99%). Data are presented as mean ± SD

All antibodies were purchased from R&D Systems (Minneapolis, MN, USA). Isolated B cells in the previous step were stained with mouse anti-human CD3e PE-conjugated monoclonal antibody (mAB) and either mouse anti-human CD19 fluorescein-conjugated mAB or mouse IgG_1_ fluorescein isotype control. The samples were read by the Cyflow Space flow cytometer (Partec, Germany), and data were analyzed by Flomax software (Partec, Germany). B cell purity was more than 80%, as measured by CD19 expression (Fig. 5).

### Gene expression assay

Total RNA was extracted from isolated B cells by an RNA extraction kit (Pars Toos: Iran) and converted into cDNA through a cDNA synthesis kit (Pars Toos, Iran) with the random hexamer primer and oligo (dT). The reverse transcription process was performed according to the one-step protocol recommended by the mentioned manufacturer: 25 °C for 10 min, followed by 47 °C for 60 min, and the reaction was finished by 85 °C for five minutes, and finally, the microtubes were chilled on the ice. Moreover, the RT-PCR technique was performed by $\frac{50 ng}{20\mu L}$ of the synthesized cDNA (as template), specific forward and reverse primers (concentration of the primers: 0.5 μM), high ROX SYBR Green Master Mix (Takara, Japan), and nuclease-free water using an Applied Biosystems RT-PCR system (ABI, StepOnePlus software, USA) with this program: 1 cycle of 95 °C for 15 min, 40 cycles of 95 °C for 5 s, and adjusted annealing temperature for 40 s. Notably, the melting curve protocol was also proceeded later by 10 s at 95 °C and then 10 s each at 0.2 °C enhancements between 62 and 95 °C. The sequences of the target and the reference genes are shown in Table 4. The RT-PCR was performed in triplicate, and β-actin was utilized as the reference gene to normalize the target genes’ amplified signal. Moreover, the relative expression of the RT-PCR products was equally calculated via the 2^-ΔΔCt^ formula. The dissociation phases, melting curve, and the quantitative analysis of data were also completed with the StepOnePlus® software version 2.3 (Applied Biosystem, Foster City, CA, USA).

**Table 4:** The sequences of primers used in the study

Gene	Forward	Reverse
Actin β	GCGCGGCTACAGCTTCA	CTTAATGTCACGCACGATTTCC
EBI3	TTCGTGCTTTCATAACAGAGCACATCA	CTCCAGTCACTCAGTTCCCCGTAGTCTG
IL-12p35	ACATGCTGGCAGTTATTGATGA	TGAAGAAGTATGCAGAGCT
IL-10	TTACCTGGAGGAGGTGATGC	GGCCTTGCTCTTGTTTTCAC
TGF-β	CCACCTGCAAGACCATCGAC	CTGGCGAGCCTTAGTTTGGAC
PDL-1	AAACAATTAGACCTGGCTG	TCTTACCACTCAGGACTTG
FasL	CTTGGTAGGATTGGGCCTGG	TGTGTGCATCTGGCTGGTAG

*EBI3* Epstein-Barr Virus Induced 3, *TGF-β* transforming growth factor beta, *PDL-1* programmed death-ligand 1, *FasL* Fas ligand

### Statistical analyses

GraphPad Prism 7.03 (GraphPad Software, San Diego, CA) and the IBM SPSS 20 (SPSS; Inc.; Chicago; IL: USA) were used to fulfill statistical analysis. The one-sample Kolmogorov–Smirnov and Shapiro–Wilk tests further assessed the normality of the variables. Differences between the studied groups were also evaluated by Chi-square statistic, independent sample T-test, Mann–Whitney U test, one-way ANOVA (Tukey’s multiple comparisons), and Kruskal–Wallis tests. Moreover, the Spearman correlation test was used to verify the correlation between disease activity and serum levels of autoantibodies and cytokines, as well as inhibitory molecules. The principal component analysis (PCA) was used to explore the obtained data from the studied cytokines [31]. Hierarchical Clustering on Principal Components (HCPC) function from the FactoMine R package was employed to apply an HCA-based on principal component scores estimated from PCA analysis. The Factoextra package was also used to visualize a dendrogram generated by the hierarchical clustering. In addition, a principal component regression (PCR) test was used to explore the relationship between disease activity (SLEDAI) and cytokines adjusted by auto-antibodies (as potential confounders). Due to the low number of SLE patients, we used a bootstrap technique to inference regression coefficients [32]. All data are presented as mean ± SD. A *p*-value of less than 0.05 was considered statistically significant.

## Data Availability

The datasets used and/or analyzed during the current study available from the corresponding author on reasonable request.

## Abbreviations

Bregs: Regulatory B-cells
SLE: Systemic lupus erythematosus
ACR: American College of Rheumatology
IL: Interleukin
ELISA: Enzyme-linked immunosorbent assay
TGF-β: Transforming growth factor beta
PD-L: Programmed death ligand 1
PD-1: Programmed death 1
UV: Ultraviolet light
ds-DNA: Double strain stranded deoxy ribonucleic acid
Sm Ag: Smith antigen
RNPs: Ribonucleoproteins
ANA: Anti-nuclear antibody
T2-MZP: Transitional 2-marginal zone precursor
RA: Rheumatoid arthritis
pDCs: Plasmacytoid dendritic cells
IFN-α: Interferon alpha
Th: T helper
NK: Natural killer
SLEDAI: Systemic lupus erythematosus disease activity index
DoH: Declaration of Helsinki
NWF: Nylon wool fiber
PBMC: Peripheral blood mononuclear cell
PCA: Principal component analysis
HCPC: Hierarchical clustering on principal components
PCR: Principal component regression
FACS: Fluorescence-activated cell sorting
